# Differential aggregation properties among Baltic Sea picocyanobacterial strains impact their export

**DOI:** 10.1093/ismejo/wrag214

**Published:** 2026-08-27

**Authors:** Martin Ekman, Elina Viinamäki, Anders Verstraaten-Svensson, Ryan W Paerl, Anders F Andersson, Helena Höglander, Jakob Walve, Rachel A Foster

**Affiliations:** Department of Ecology, Environment and Plant Sciences (DEEP), Stockholm University, SE-106 91 Stockholm, Sweden; Department of Clinical Microbiology, Karolinska University Hospital, SE-14186 Stockholm, Sweden; Department of Ecology, Environment and Plant Sciences (DEEP), Stockholm University, SE-106 91 Stockholm, Sweden; Department of Ecology, Environment and Plant Sciences (DEEP), Stockholm University, SE-106 91 Stockholm, Sweden; Department of Marine, Earth, and Atmospheric Sciences, NC State University, Raleigh NC 27695, United States; Department of Gene Technology, Science for Life Laboratory, KTH Royal Institute of Technology, SE-100 44 Stockholm, Sweden; Department of Ecology, Environment and Plant Sciences (DEEP), Stockholm University, SE-106 91 Stockholm, Sweden; Department of Ecology, Environment and Plant Sciences (DEEP), Stockholm University, SE-106 91 Stockholm, Sweden; Department of Ecology, Environment and Plant Sciences (DEEP), Stockholm University, SE-106 91 Stockholm, Sweden

**Keywords:** picocyanobacteria, aggregation, sedimentation, 16S rRNA, Baltic Sea, colony

## Abstract

Picocyanobacteria contribute significantly to carbon export but the mechanism by which their biomass is transported to depth is unclear. By analyzing 16S rRNA gene sequences from sediment trap material and size-fractionated water samples from different depths in the Baltic Sea, we show that picocyanobacterial contribution to phytoplankton biomass export was largely proportional (70%–90%) to their surface water abundances. However, we also found that both cell aggregation (enrichment in >10 μm fraction) and sedimentation (trap enrichment) varied among *Synechococcus* ASVs (amplicon sequence variants). A robust linear relationship was identified between ASV enrichment in traps and ASV enrichment in the >10 μm fraction, suggesting that cell aggregation is a direct determinant of *Synechococcus* sedimentation. Seasonal abundance patterns were similar among the dominant aggregate-forming ASVs but distinct from those of single-cell ASVs, suggesting aggregate/colony formation being an important trait in determining distribution of *Synechococcus* strains and their response to environmental conditions. Correlating microscopy- and sequence-based abundances further allowed us to propose genotypes for the dominant Baltic Sea colonial picocyanobacterial morphotypes. Our results provide *in situ* data linking phenotypic characteristics of picocyanobacteria with sedimentation properties and several lines of evidence for a quantitative dependency between surface water picocyanobacterial aggregate/colony formation and sinking. These findings are important for our understanding of mechanisms involved in export of pico-sized phytoplankton biomass from the photic zone to depth and emphasize the need to investigate occurrence and environmental regulation of cell aggregation in other picophytoplankton-dominated waters (e.g. open ocean gyres).

## Introduction

Phytoplankton are the primary component of the “biological pump” that ultimately removes CO_2_ from the atmosphere for export to the deep ocean by sedimentation. Among the primary producers, picocyanobacteria (Pcy), namely, small (<2 μm cell diameter) unicellular cyanobacteria belonging to the genera *Prochlorococcus* and *Synechococcus* (i.e. *Synechoccales sensu stricto* [1]), constitute the dominant group [2–4]. Although sinking of large phytoplankton (e.g. diatoms) have conventionally been considered the major component of carbon (C) export [5], more recent studies have shown that smaller cell diameter phytoplankton, including Pcy, can significantly contribute to export production [6–15]. Additionally, genetic analyses of sediment trap materials have identified differential contribution among picophytoplankton groups to C export and that *Synechococcus,* in particular, can constitute a major component of the biomass that sinks [10, 13, 14, 16, 17]. The characteristics that allow *Synechococcus* to sediment more than other small-sized phytoplankton (e.g. *Prochlorococcus*) has not been studied in detail.

The physics of settling (e.g. Stokes law) predicts that Pcy as single cells are too small to sediment through the water column and by convention are too small to be efficiently grazed by mesozooplankton [18, 19]. Hence, the mechanism for the recent reports of picophytoplankton export is unclear. Some speculate export could be mediated via formation of colonies or aggregates that are large enough to sink and/or consumption of these colonies by mesozooplankton that produce sinking fecal pellets [6, 9]. Colony formation by Pcy has been well documented in freshwater and brackish waters, where colony-forming Pcy can constitute the majority of total Pcy biomass [20, 21]. Less data are available from the oligotrophic ocean; however, high abundances of colonial/aggregating Pcy have been reported regionally [22], in global surveys (e.g. *Tara* Oceans) [23], and in lab-based studies [24–26]. To date, there has been no direct field observations or measurements on the extent to which Pcy cell aggregation impact sedimentation from surface waters.

*Synechococcus* cell aggregation may be induced in response to external stimuli [20, 25–27], and evidence from wild Baltic Sea and freshwater strains show phenotypic plasticity, allowing them to occur either as single cells or as colonies [27, 28]. However, other studies analyzing size-fractionated water samples (>10 μm, 0.2 μm) from the Baltic Sea [29] and the California Current [30] identified distinct genetic profiles of *Synechococcus* populations from different size fractions, suggesting that the tendency to occur in colonial/aggregate forms varies among *Synechococcus* strains. Recent works have also provided evidence that sedimentation may vary among *Synechococcus* strains [13, 14]. No experimental data, however, are available explaining differential sedimentation among picocyanobacterial strains or identifying phenotypic characteristics that determine a tendency of phylotypes to sediment.

The primary aim of the present study was to investigate the effect of colony formation/aggregation on Pcy sedimentation. Although colony formation and cell aggregation may be considered different processes [31], in this study, we do not attempt to differentiate them and assume that their impact on export processes is the same. Our study site was the long-term monitoring site Landsort Deep (BY31) in the central Baltic Sea, which, for >30 years, has been routinely sampled (22 times per year) by the Swedish marine monitoring program. In these waters, Pcy mostly belong to the genus *Synechococcus* [32, 33] and can constitute over 50% of total phytoplankton biomass during summer months [21, 34]. Colonial Pcy may furthermore reach up to 70% of the Pcy population [21], thus making the Baltic Sea a suitable model system to investigate sedimentation of such morphotypes. By using a combination of microscopy, flow cytometry, and 16S rRNA gene sequencing of size-fractionated water samples and sediment trap materials, we studied the effect of cell aggregation on the contribution of Pcy to particulate C export.

## Materials and methods

### Sampling

Samples were collected in parallel with the Swedish National Marine Monitoring Program every second week from 23 May to 11 September 2018 at the Landsort Deep station in the northern part of the western Baltic Proper (BY31: 58°35.9′N, 18°14.2′E) (Fig. S8). Salinity and temperature profiles were obtained from standard conductivity, temperature, and depth (CTD) casts (Memory probe by Sea & Sun Technology GmbH). The depth of the photic zone was calculated from Secchi depth measurements and a conversion factor of 2.7 [35]. Discrete water samples were collected with serial Niskin bottles from 10 depths for analysis of nutrients and chlorophyll *a* (*chla*) as described elsewhere [36, 37]. In addition to the discrete depth sampling, a parallel depth integrated (0–20 m; hereafter DI) water sample (20 L) was collected with a 20 m hose deployed from the side of the bow, for identification and quantification of phytoplankton. A subsample (250 ml) was collected from the DI sample and fixed with acidic Lugols solution (1–250 ml sample). The resulting chemical/physical data and experimental procedures are part of the monitoring program and are available at https://sharkweb.smhi.se/hamta-data/ (Manual—HELCOM, 2017) [37] and as Suppl. Data File 1. Sediment traps were deployed as is detailed below.

#### Samples for DNA analysis

Ten-liter water samples were collected from three depths: DI, 25 m, and 75 m. The latter two depths were collected with a Niskin bottle attached to a hydro wire. Triplicate whole water samples (175–500 ml) for DNA extraction were collected from the three depths by two different filtration procedures using a peristaltic pump (Cole-Parmer, Vernon Hills, IL, USA): (i) directly filtered onto a 0.2 μm pore size Supor filter (Pall Corporation) held in a 25 mm diameter filter holder (Millipore, Billerica MA, USA), which represents the total community, and (ii) size-fractionated by serially filtering through two 25 mm diameter filters: 10 and 0.2 μm pore size filters in line to separate picocyanobacterial colonies/aggregates from single cells. In our samples both single-cell and the dominant colonial Pcy had cell diameters around 1 μm (Fig. S3), which is consistent with previously reported Pcy cell diameters from the Baltic Sea [32, 38]. Typically, during the serial filtration, after 500 ml, the 0.2 μm filter clogged and was removed, and subsequently, the remaining 1.5 L was filtered only onto the 10 μm pore size filter. After filtration, filters were folded in half, flash-frozen in liquid N_2_, and stored at −80°C. In addition to the water samples, sediment trap material was collected. Sediment traps [39, 40], each with two exchangeable sampling cylinders (ø 105 mm, length 500 mm) were moored below the thermocline at ~25 m depth. One of the cylinders contained 1 L of brine solution (sodium chloride 33.8 g/L, magnesium chloride, 14.3 g/L, potassium chloride 0.93 g/L) and the other 0.1% chloroform to allow comparison of “live” versus fixed traps. However, as only limited differences were seen between the two types of traps, they were instead considered replicates. Approximately every 2 weeks after deployment, the traps were recovered, returned to the laboratory, and left in a 4°C room overnight to re-settle. The contents were filtered with a peristaltic pump onto 0.2 μm filter cartridge (Sterivex, Millipore Sigma, Burlington, MA, USA) and frozen in liquid N_2_. When possible, the entire contents of the trap were filtered onto a sterivex filter; however, often, several sterivex filters were required due to a high amount of particles (Table S1). Trap material was only used for DNA analysis.

#### Samples for flow cytometry analysis

Each of the three water depths were subsampled in triplicate for flow cytometry analyses; 4.6 ml of collected water was added to a 5 ml cryotube containing 400 μl of 25% glutaraldehyde solution (v/v), immediately flash-frozen in liquid N_2_ and stored at −80°C. Samples were transported to the analytical facility (see below) for analyses in a dry shipper to ensure −80°C conditions.

#### Phytoplankton long-term trends

In addition to the 2018 time series sampling described above, we also analyzed long-term trends (2013–2022) in phytoplankton abundances. These data were downloaded from a public repository (https://sharkweb.smhi.se/hamta-data/) that contains over 30 years of Baltic Sea monitoring data and is maintained by the Swedish Government agency Swedish Meteorological and Hydrological Institute (SMHI). All raw data downloaded from this depository are available as Supplementary Data Files 1 and 2. Procedures for collection and analysis of these samples are the same as those described for the 2018 time series.

### Microscopy analysis of phytoplankton abundance and community composition

The abundance of surface (0–20 m) phytoplankton taxa, including colony-forming picocyanobacteria, was determined in the 250 ml subsample from the DI sample by the Utermöhl method [37]. The Utermöhl method relies on settling of phytoplankton and largely excludes phytoplankton smaller than ~2 μm, such as single-cell Pcy and also small Pcy colonies and aggregates (<10 cells/colony). Phytoplankton (>2 μm) were counted after sedimentation in 10 or 25 ml chambers using an inverted microscope with phase contrast. Microphytoplankton (including colony-forming picocyanobacteria) were counted on the half/whole chamber bottom or in diagonals with a 10× or 20× objective (total magnification 100× or 200×, colony-forming picocyanobacteria in 200×). Nanoplankton were counted in two diagonals with a 40× objective (total magnification 400×). Phytoplankton, including colonial Pcy, were classified taxonomically based on morphology as previously described (HELCOM 2017) [37] (Fig. S3). Biomass (biovolume and carbon) of phytoplankton cells were calculated as described previously [41] and the HELCOM taxa-specific biovolume and carbon table at www.ices.dk/data/Documents/ENV/PEG_BVOL.zip (used version PEG_BVOL2018) (Suppl. Data File 3). Colonial Pcy included three dominant morphotypes, classified based on morphology as *Cyanodictyon*, *Aphanocapsa,* and *Aphanothece* for which cell diameters were 1.0 × 1.2 μm, 1.2 × 1.2 μm and 1.5–2.0 × 0.8–1.0 μm, respectively (Fig. S3). As the 16S rRNA gene analysis annotate these organisms to *Synechococcus/Cyanobium,* we choose to refer to these morphotaxa as “morphotype 1–3.” Data on phytoplankton abundance and biomass were obtained from https://sharkweb.smhi.se/hamta-data/ and are available as Suppl. Data File 2.

### Flow cytometry analysis of picocyanobacterial abundance

The Pcy abundance (including single cells) were analyzed by flow cytometry at North Carolina State University (NCSU) using a Millipore Guava easyCyte HT system (Luminex Corporation) (blue excitation: Yellow-Blue = 583/26 nm, Green-Blue = 525/30 nm, Red-Blue = 695/50 nm and red excitation: Red-Red = 662/15 nm). A maximum of 30 000 events were counted per sample. Counts per sample were determined from pooled counts from six wells (300 μl sample each). Data were analyzed using InCyte software (Millipore). Total picocyanobacterial counts were determined using fluorescence from the blue excitation laser (Yellow-Blue = 583/26 nm; fluorescence typical of phycoerythrin (PE) and PE-like pigments [42], and red fluorescence from the blue excitation laser (Red-Blue = 695/50 nm; typical of chlorophyll a). Reference beads with a known diameter were used to convert forward scatter values for unknown events (Pcy cells) to μm diameter. Pcy populations were divided into three size classes; single cells (diameter 0.5–2 μm), microcolonies (diameter 2–3 μm), and colonies (diameter > 3 μm) (Fig. S9a and b). To verify the classifications, cells/colonies from the three different size classes were sorted using a BD FACS Melody 3-laser 9-color detection system (NCSU MicroFACS Facility). Sorted cells were examined using a Nikon Eclipse TsR2 inverted microscope [SOLA light engine (LED)] using epifluorescence in a darkened room (Fig. S9c–e). For details on using the flow cytometry (FCM) counts to calculate total and single-cell Pcy cell concentrations and biomass, see Supporting information.

### DNA extraction, amplification, sequencing, and data analysis

DNA extraction of samples collected on 10 and 0.2μm filters were carried out using the DNeasy PowerWater Kit (Qiagen) according to the manufacturer’s instructions. DNA extraction of samples collected on Sterivex filters (trap samples) were carried out using the DNeasy PowerWater Sterivex Kit (Qiagen) according to the manufacturer’s instructions. Extracted samples were eluted in 100 μl EB buffer (Qiagen), normalized, and sent to the National Genomics Infrastructure/Science for Life Laboratory (Stockholm, Sweden) for amplification and sequencing. The V3–V4 region of the 16S rRNA gene was amplified using the 341F/805R primers [43, 44] using a previously described protocol [45]. Amplicon sequencing was performed on a MiSeq System (Illumina), resulting in, on average, 80 000 2 × 300 bp paired end reads per sample. Sequence data were processed using the nf-core/ampliseq pipeline [46, 47] with software version 1.1.2 and taxonomically classified using SILVA 138.2. Chloroplast sequences were re-classified using the PhytoREF sequence database [48]. Raw reads have been deposited in NCBI under BioProject ID PRJNA1184790 and Biosample Accession Nos SAMN44675630-715.

From each trap (carrying two cylinders), two to six replicate sub-samples were sequenced, whereas for water samples, only one of the three replicates collected for each sample category (size fraction/depth/date) was sequenced (Table S1). From sediment trap replicates, average relative abundances were calculated using the relative abundances determined for each replicate. The nonsize-fractionated DI sample from 2 July was lost, and relative abundances were estimated by combining data from the two size fractions from the same sample as described previously [30] (see also Supporting information, and Fig. S10). For phylogenetic analyses, sequences were aligned using the MUSCLE algorithm and maximum-likelihood (ML) phylogenies were constructed using the MEGAX software. A total of 500 bootstrap replicates were conducted. Trees were visualized in Archeopteryx.

## Results

### Physical/chemical conditions over the sampling season

Physical/chemical conditions during the sampling (May–September) followed the seasonality expected at BY31 (Fig. S1). The sea surface temperature (0–10 m) was highest in late July, when a shallower thermal stratification had re-established after a deeper mixing in early July. Surface concentrations of dissolved inorganic phosphorus (DIP) were higher (0.063 μmol L^−1^) in the beginning of the season (May), largely depleted through summer (June to mid-August) due to uptake by the surface planktonic community, and again elevated later in the season (27 August; 0.076 μmol L^−1^) after remineralization. Similarly, ammonia increased in late July, likely due to the increase in abundance of N_2_-fixing cyanobacteria and expected release of reduced nitrogen (N) (see below).

### Phytoplankton abundance in surface waters during the sampling season by microscopy and flow cytometry analyses

Phytoplankton abundance in the DI (0–20 m) samples varied over the season both in terms of biomass (reported as C concentration, μg L^−1^) and extracted chl *a*. The highest values for both variables were recorded in mid-June, and a second peak in chl *a* was measured during mid-July (Fig. 1a). Over the season eukaryotic phytoplankton (>2 μm) contributed most to total biomass (40%–85%), whereas N_2_-fixing filamentous cyanobacteria (*Aphanizomenon, Nodularia,* and *Dolichospermum*; hereafter *Nostocales*) and Pcy (including colonies and single cells) constituted at most 36% and 49%, respectively (Fig. 1a). Other cyanobacteria made up at most 1.7% of phytoplankton biomass in any sample (Fig. S2a). In terms of cell counts, Pcy dominated the phytoplankton community throughout the sampling season (>97%) (Fig. S2b).

**Figure 1:**
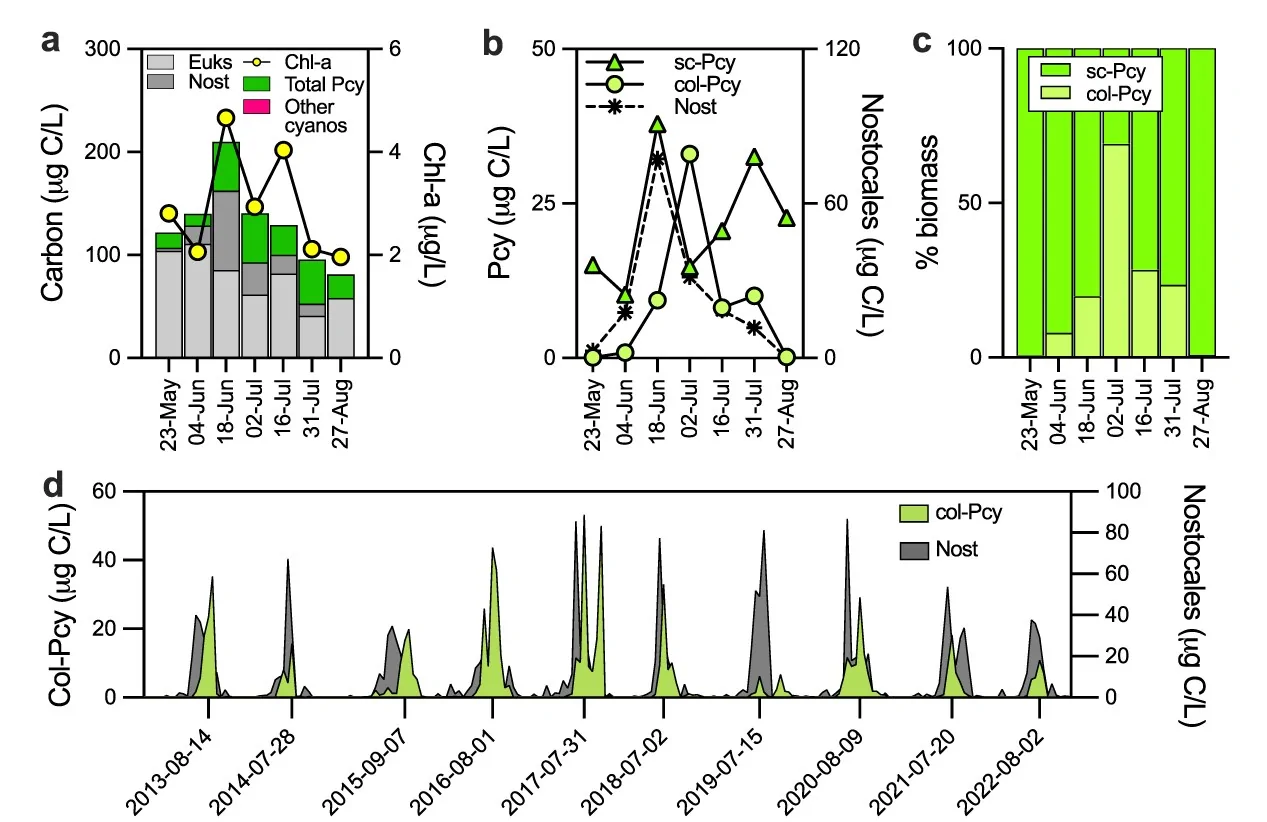
Seasonal trends in Baltic Sea phytoplankton identified by microscopy and flow cytometry. (a) Chlorophyll a (mg Chl a/L) (yellow circles) and biomass (μg C/L) (bars) for eukaryotic phytoplankton (Euks) (light gray), *Nostocales* (Nost; *Aphanizomenon* sp*., Nodularia spumigena,* and *Dolichospermum* spp.) (dark gray), picocyanobacteria (Total Pcy) (green), and other cyanobacteria (other cyanos; *Pseudanabaena, Planktolyngbya, Snowella*, and *Woronichinia*) (pink). Picocyanobacteria represent the total population (both colonial and single-cell forms). Chlorophyll a is average value of samples collected from 0, 5, 10, 15, and 20 m. (b) Seasonal trends of colonial Pcy (col-Pcy, light-green circles), single-cell Pcy (sc-Pcy, dark-green triangles), and *Nostocales* (Nost, black asterisks, ^*^). (c) sc-Pcy (dark green) and col-Pcy (light green) biomass as percentage of total Pcy biomass. (d) Seasonal trend in biomass of *Nostocales* (Nost, gray area) and col-Pcy (light-green area) during the years 2013–22 at BY31. Dates are peak abundance (biomass) for col-Pcy each year. The raw data for (d) originate from the Baltic Sea Monitoring Program and are available as Suppl. Data File 2 or from https://sharkweb.smhi.se/hamta-data/. All abundances (biomass) were based on microscopy data, except for Total Pcy that was based on FCM data and single-cell Pcy that was estimated from combining FCM and microscopy data (see Materials and Methods).

A pronounced increase in colonial Pcy biomass and cell densities was obvious on 2 July, whereas single-cell Pcy showed a distinct peak in abundance 2 weeks earlier, which coincided with peak abundance of *Nostocales* (Figs 1b, S2c and d, and  S3), and a second peak at the end of July. The contribution of colonial Pcy to the total Pcy population varied over the season and reached its highest value (60%) in early July (Fig. 1c, Fig. S2e). The observed succession from *Nostocales* to colonial Pcy is frequently seen in the preceding 10 years of monitoring data (Fig. 1d, for finer taxonomic resolution, see Fig. S2f).

### Phytoplankton representation in water samples and traps by 16S rRNA gene sequence analyses

Consistent with both the microscopy observations and FCM (Fig. 1a), phytoplankton 16S rRNA gene sequences were throughout the sampling season and in all sample categories, mainly (99.4% of phytoplankton sequence reads) annotated as chloroplasts (i.e. eukaryotic phytoplankton), *Nostocales,* and the picocyanobacterial *Synechococcales* genera *Synechococcus/Cyanobium* (hereafter Syn) (Fig. 2a, Fig. S4a). Additionally, sequences from other cyanobacteria groups (*Leptolyngbyales, Phormidesmiales, Pseudanabaenales*; <0.6% of phytoplankton sequences) and heterotrophic bacteria, were recovered. Syn dominated among phytoplankton sequences at each sampling occasion in all nonsize-fractionated DI samples (77%–92%), the discrete depths: 25 m (70%–96%) and 75 m (61%–89%), and additionally in all trap samples (54%–91%) (Fig. 2b, Fig. S4a).

**Figure 2:**
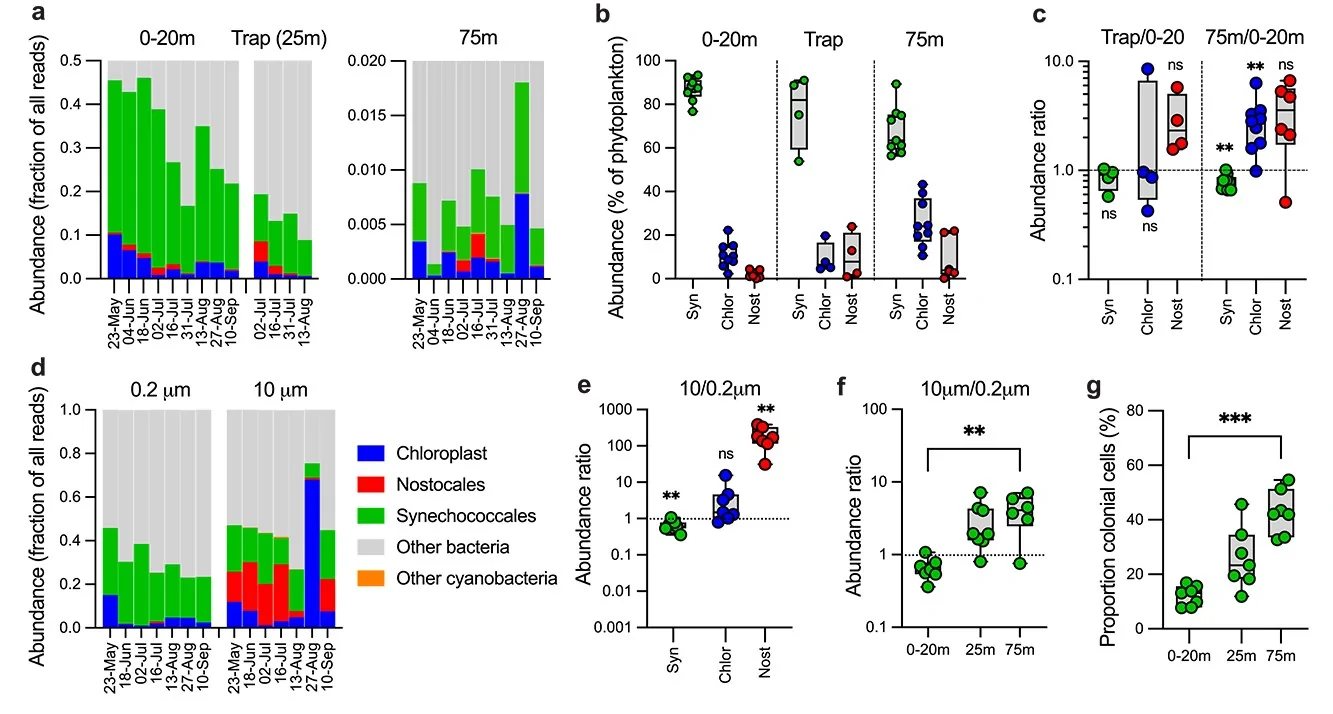
Abundance of the major phytoplankton groups at different depths, in traps and in different size fractions. (a) Overall seasonal 16S rRNA gene sequence-based community composition in nonsize-fractionated 0–20 m depth-integrated (DI), sediment trap (25 m), and 75 m samples. “Chloroplasts” indicate eukaryotic phytoplankton. (b) Box plot showing abundance of phytoplankton groups as percent of total phytoplankton sequences, in all nonsize-fractionated DI, trap, and 75 m samples. (c) Box plot showing ratio between the abundance (as percent of total phytoplankton reads) of phytoplankton groups in traps vs DI samples and in 75 m vs DI samples. (d) Phytoplankton representation in the two serial size fractions of DI samples. (e) Box plot of ratios between abundance of phytoplankton groups (relative to all reads) in the two size fractions of DI samples. (f) Box plot of ratios between *Synechococcales* abundance (relative to all reads) in 10 vs 0.2 μm size fractions. Size fraction ratios are displayed according to sampling depth. (g) Box plot for flow cytometry (FCM) data of colonial picocyanobacteria (Pcy) as percentage of total Pcy counts in water samples from different depths. All box plots show median and interquartile range. Differences in relative abundance (c and e) or average ratio (f and g) were evaluated using Student’s *t-*test. ns = not significant (*P* > .05). Significant differences are shown as: ^*^P < .05, ^**^P < .01, ^***^P < .001. The color key panel applies to panel (a–f). In panel (g), green is for colony-forming Pcy.

Average sediment trap representation did not differ significantly from that of the DI samples for any of the three main phytoplanktonic groups (Fig. 2c). At 75 m, the Syn component of the phytoplankton community was smaller than in surface waters [average ratio 0.79 ± 0.11(SD)], whereas “chloroplasts” made up a correspondingly larger component [average ratio 2.8 ± 1.5 (SD)] (Fig. 2c). The *Nostocales* representation in 75 m samples was not statistically different from the DI samples. However, in comparison to Syn the representation of both chloroplasts and *Nostocales* was significantly higher at 75 m (Anova, *P* = .0014).

In the size-fractionated DI water samples, Syn made up a considerable component of 16S rRNA gene reads in not only the small (0.2–10 μm) fraction (19%–37%) but also in the large (>10 μm) size fraction (6%–23%) (Fig. 2d). Compared to the small size fraction, Syn was underrepresented in the large size fraction whereas chloroplasts and especially *Nostocales* were overrepresented (ANOVA, *P* < .0001) (Fig. 2e). However, the large size fraction representation increased with depth for Syn, and at both 25 and 75 m, Syn was overrepresented in the large compared to the small size fraction (Fig. 2f). The latter trend was consistent with the ratio of abundances determined by FCM (Fig. 2g); from the surface (0–20 m DI samples) down to 75 m, colonial Pcy significantly increased from an average of 12% to an average of 42% of the total Pcy population.

### Diversity of *Synechococcus* in water samples and traps

In the entire dataset (including both fractionated and nonfractionated samples) >99.9% of *Synechococcales* sequence reads were annotated to *Synechococcus*/*Cyanobium*, for which 55 amplicon sequence variants (ASVs) were identified. The following analyses focused on the 17 most abundant ASVs that collectively contributed to >99% of all Syn 16S rRNA gene reads in the dataset (Fig. 3a and f, Fig. S4c and d).

**Figure 3:**
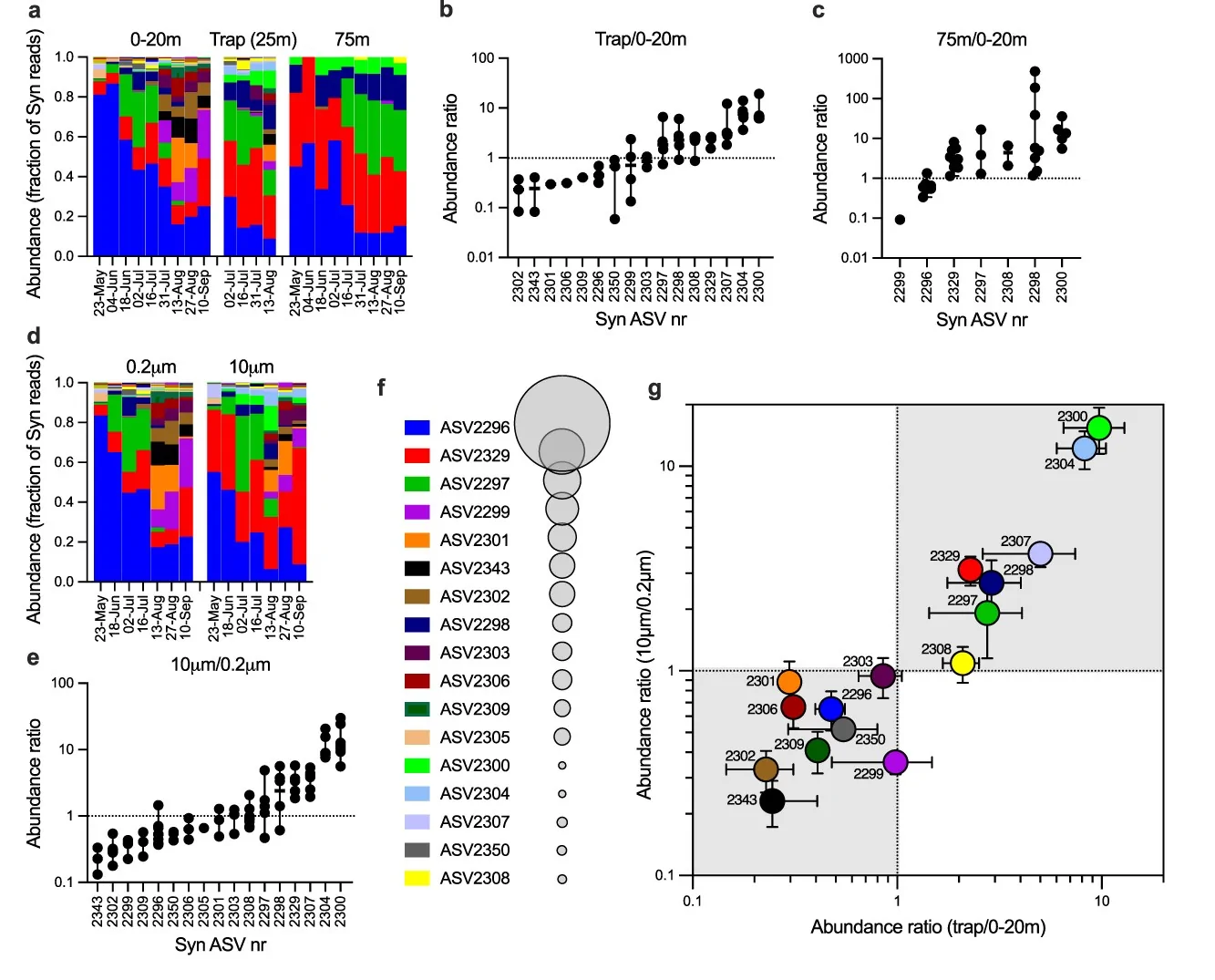
Distribution of *Synechococcus/Cyanobium* (Syn) ASVs at different depths, in traps and in different size fractions. (a) Abundance of Syn ASVs (as fraction of all Syn reads) in nonsize-fractionated 0–20 m depth-integrated (DI), sediment trap (25 m), and 75 m samples. (b) Ratios between the relative abundance of individual ASVs in traps and DI samples collected on the same day. (c) Ratios between the relative abundance of individual Syn ASVs in 75 m and DI samples collected on the same day. (d) Abundance of Syn ASVs (as fraction of all Syn reads) in the 10 and 0.2 μm size fractions of serially filtered 0–20 m DI samples. (e) Ratios between the relative abundance of individual Syn ASVs in the 10 μm and 0.2 μm size fractions of serially filtered DI samples. (f) Median relative abundance of Syn ASVs in all 0–20 m non-size fractionated DI samples shown as circles. (g) Correlation between the average trap/DI and 10 μm/0.2 μm ratios for individual Syn ASVs. Shaded areas indicate ASVs that are either overrepresented (ratios >1), or underrepresented (ratios <1) in both traps and the 10 μm size fraction. Error bars represent SEM. The ASV abundance ratios (trap/DI and 10 μm/0.2 μm) show significant positive correlation (*R*^2^ = 0.93, *P* < .0001). The color code in panel (f) is for panels (a), (d) and (g). All relative abundances were calculated as fraction of all Syn reads.

Compared to DI samples, certain Syn ASVs were consistently overrepresented in sediment traps (ratio > 1) and others were consistently underrepresented (ratio < 1) (Fig. 3b, Fig. S4c). A similar trap representation of ASVs was obtained when comparing with the 25 m samples (Fig. S5a). At 75 m, fewer ASVs were detected but those overrepresented at this depth (relative to DI samples) were all also overrepresented in traps, and likewise, ASVs underrepresented at 75 m were also underrepresented in traps (Fig. 3c). At 25 m, differences in representation (relative to DI samples) among ASVs were less pronounced and only partly matched that of traps and 75 m samples (Fig. S5b).

Comparing the Syn ASV representation in the two size fractions of DI samples (Fig. 3d) showed that certain ASVs were throughout the sampling season consistently overrepresented in the large size fraction, whereas others were consistently underrepresented (Fig. 3e, Fig. S5c). At 25 and 75 m depths, both size fractions were dominated by ASVs overrepresented in the large fraction of DI samples (Fig. S4c). Furthermore, the ASV representation in traps and the large size fraction show a strong positive correlation (*R*^2^ = 0.91, *P* < .0001) (Fig. 3g). In contrast, representation at 25 m correlated poorly with trap representation (*R*^2^ = 0.023, *P* = .619) (Fig. S5d). For brevity, the seven ASVs overrepresented in the large fraction (and traps) (i.e. 2297, 2329, 2298, 2300, 2304, 2307, and 2308) will from here on in the text be referred to as “colonial” and ASVs underrepresented in these samples as “single cell.”

### Consistent seasonal trends in colonial and single-cell *Synechococcus* by microscopy and ASV analyses

According to microscopy observations the colonial Pcy population was dominated by three morphotypes, that via morphological features were classified as *Cyanodictyon, Aphanocapsa,* and *Aphanothece* (Fig. S3). However, as no 16S rRNA gene sequences were annotated to these taxa, we conclude that the three colonial Pcy morphotypes genetically must be represented among the ASVs annotated to *Synechococcus/Cyanobium* (Syn ASVs), which is in agreement with previous reports from the Baltic Sea [38, 49]. For simplicity we will hereafter refer to these microscopy classified colonial Pcy as “Morphotypes 1–3” (Fig. S3). These three morphotypes together composed >98% of all colonial Pcy from May until the end of July with the highest abundances observed on 2 July (Fig. 4a). In particular, Morphotype 1 had a very pronounced abundance maxima on 2 July (1.32 × 10^8^ cells/L), which represented 77% of the total colonial Pcy population. In comparison, the three most abundant colonial Syn ASVs (2297, 2329, and 2298) collectively over the season represented 94% of the total colonial Syn ASVs in nonsize-fractionated DI samples, and show the same temporal (relative) abundance patterns as the (absolute) abundance pattern of three colonial Pcy morphotypes enumerated by microscopy (Fig. 4b). Similar to colony Morphotype 1, one of the colonial Syn ASVs, ASV2297, dominated, and on 2July represented 10% of all 16S rRNA gene reads and 60% of colonial Syn ASVs (Fig. 4b, Table S2). The four-remaining colonial ASVs (2300, 2304, 2307, and 2308) showed not only the same abundance peak on 2 July but also a more pronounced presence in mid-August (Fig. S6). In contrast to the colonial ASVs, the dominant single-cell ASV2296 showed peak abundance in May and June and then gradually decreased until the end of July (Fig. 4c).

**Figure 4:**
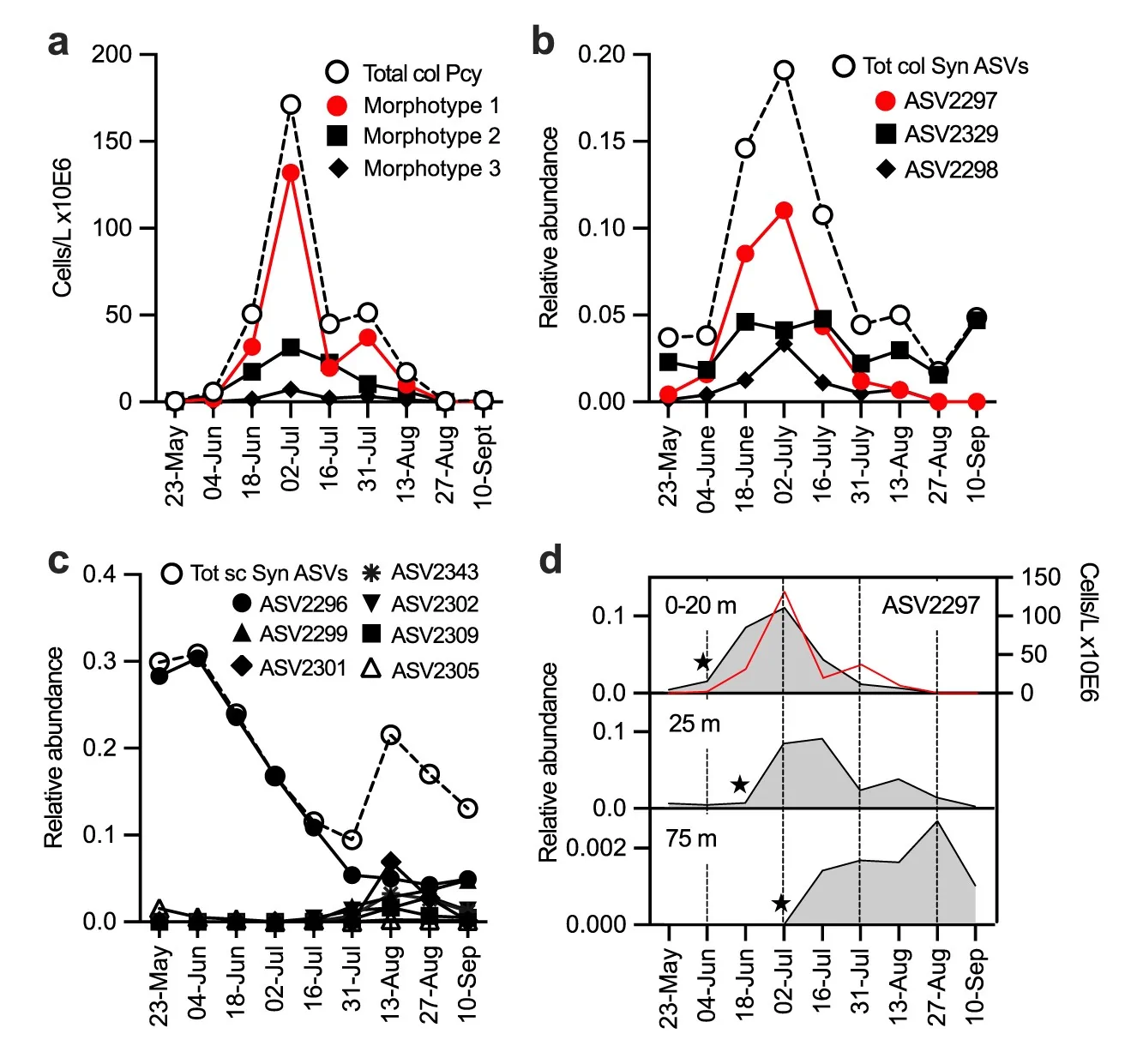
Seasonal trends in picocyanobacterial abundance based on microscopy and 16S rRNA gene sequencing. (a) Microscopy determined seasonal abundance of the three dominant colonial picocyanobacterial morphotypes and their combined abundance (Total col Pcy) in 0–20 m depth-integrated (DI) samples. (b) 16S rRNA gene sequence–based abundance (as fraction of all reads) of the three most highly abundant colonial *Synechococcus/Cyanobium* (Syn) ASVs and the combined abundance of all colonial Syn ASVs (Total col Syn ASVs) in nonsize-fractionated DI samples. (c) 16S rRNA gene sequence–based abundance (as fraction of all reads) of single-cell Syn ASVs and the combined abundance of all single-cell ASVs (Total sc Syn ASVs) in DI samples. (d) Abundance of ASV2297 (as fraction of all reads) at the three sampling depths. The red line indicates the microscopy determined seasonal abundance (right axis) of colonial Pcy Morphotype 1. Stars indicate from which timepoint ASV 2297 sequence reads are detected at the different depths.

When comparing the seasonal abundance for the dominant colonial ASV2297 at the three sampling depths, each depth showed a similar transient increase in abundance (Fig. 4d). However, for the 25 and 75 m sampling depths, abundance maxima were temporally shifted to later in the season: 2 weeks for 25 m and 4 weeks for 75 m. For the remaining five Syn ASVs detected at all three depths in the nonsize-fractionated dataset (2296, 2329, 2298, 2300, and 2308) such shifts were less clear, especially for ASVs without distinct surface water abundance peaks (Fig. S7). Depth-dependent shifts in abundance maxima were, however, observed for other phytoplankton taxa including the dominant *Nostocales* genera *Nodularia* (Fig. S7f) and *Aphanizomenon* (Fig. S7g). Among diatoms, a distinct abundance peak was observed for *Coscinodiscales* in late August (Fig. S7h). This taxon dominates among diatom sequences at 75 m (92%) but show no time lag with depth. Sequence abundances of *Aphanizomenon, Nodularia,* and *Coscinodiscales* were closely matched by microscopy observations (red line Fig. S7f–h) despite microscopy providing absolute abundances, and 16S rRNA gene sequencing relative abundances (i.e. fractions of all sequence reads).

With the assumption that the ASV2297 depth/time-shifted abundance peaks (Fig. 4d) represent sinking biomass, the sinking velocity of this phylotype was roughly estimated. Using 25 m as a “starting point” and 75 m as the “end point,” it takes ~2 weeks for the biomass of ASV2297 to sink 50 m, at a sinking velocity of 3.6 m/day. For *Aphanizomenon*, the time lag between peaks suggests a similar sinking velocity, whereas for *Coscinodiscales*, the absence of time lag suggests a higher velocity.

### Phylogeny of *Synechococcus* ASVs

Phylogenetic analysis supported all 17 of the high-abundance Syn ASVs from BY31 within the picocyanobacterial *Synechococcus/Cyanobium* clade and most of these (14 of 17, 82%) within the subcluster 5.2, which includes reference strains from coastal, brackish, and freshwater environments (Fig. 5). Two ASVs belong to subcluster 5.1 that contain reference strains from open ocean. The highly abundant single-cell ASV2296 is clustering with only freshwater strains and appears to not be closely related to any reference strain. Its 16S rRNA gene sequence is identical to the BACL30 strain [50] which in earlier studies has been shown to dominate the *Synechococcus* population in central and southern Baltic Sea [33, 51]. Of the seven colonial ASVs, one (ASV2298) is found in subcluster 5.1, whereas the remaining six are found in the 5.2 subcluster; five of these cluster with *Synechococcus/Cyanobium* reference strains and one (ASV2297) with ASV2296/BACL30. None of the three dominant colonial Syn ASVs (2297, 2329, and 2298) clustered closely with sequences annotated to *Cyanodictyon, Aphanothece,* or *Aphanocapsa.* Among these three organisms, only *Cyanodictyon* was found within the picocyanobacterial clade. Bootstrap values were in general poor, due to the limited resolution obtained by the V3–V4 16S rRNA gene sequence analyzed here.

**Figure 5:**
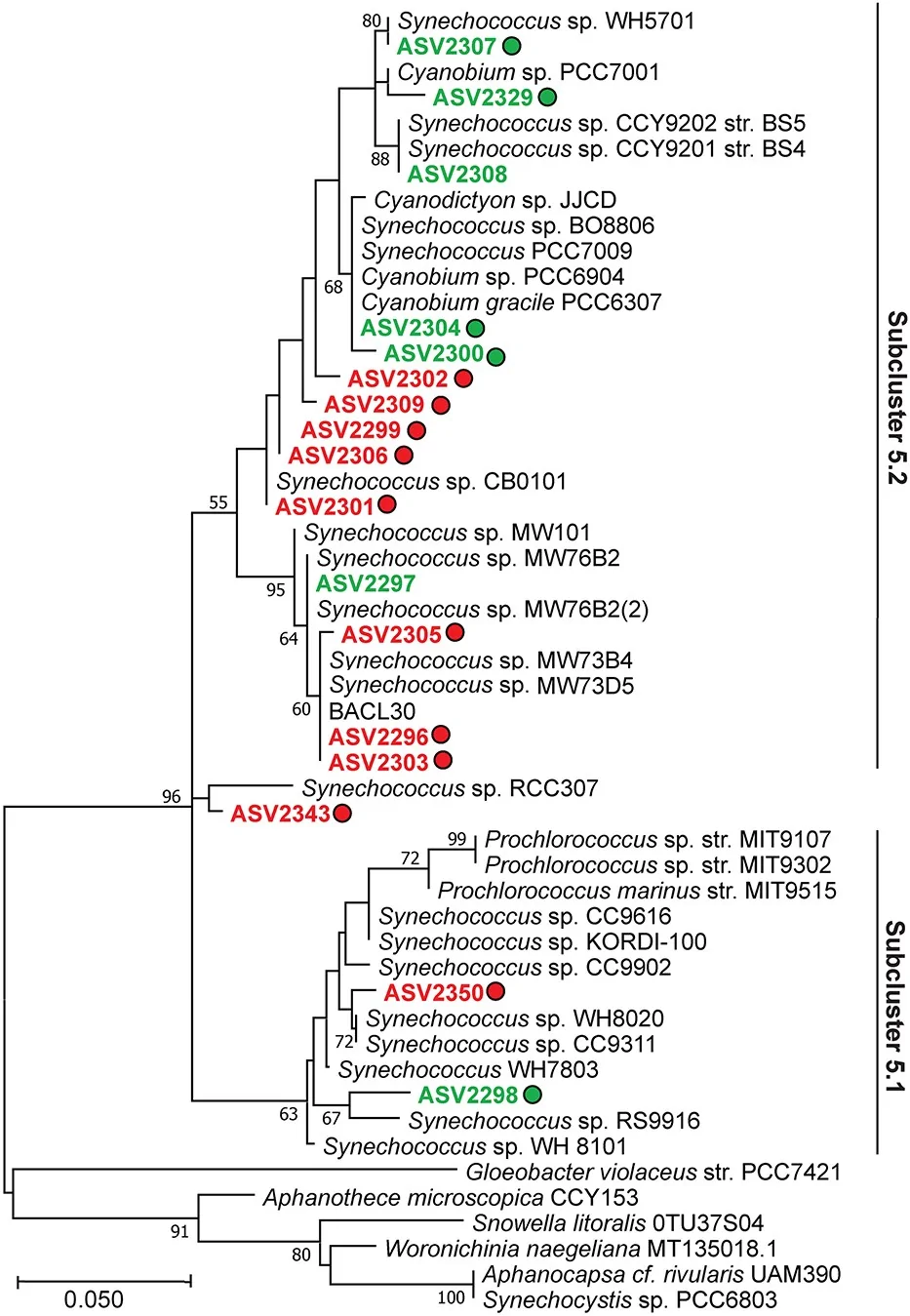
Phylogenetic analysis of the 17 most abundant picocyanobacterial 16S rRNA gene sequences from this study and a selection of reference strains. Colonial ASVs (i.e. those enriched in the >10 μm size fraction) are indicated with green dots and single-cell ASVs (i.e. those under-represented in the >10 μm size fraction) with red dots. The tree was rooted using *Gloeobacter violaceus* PCC7421. Bootstrap support (500 replicates) values above 50 are shown at nodes and the scale bar indicates number of expected substitutions per site.

## Discussion

### Seasonal trends of phytoplankton and Pcy abundance

Pcy often dominate among phytoplankton in oligotrophic regions of the oceans, but may also contribute significantly in coastal waters [42, 52, 53]. The abundances determined in the present study (3 × 10^8^ cells L^−1^) agree with previous estimates of Pcy abundance during summer months in the Baltic Sea (1–17 × 10^8^ cells L^−1^) [21, 53–55] and emphasize their ecological importance in these waters. Additionally, our estimates of Pcy contribution to total phytoplankton biomass (7%–49%) are within the range of previous reports for the area [21, 32, 34].

In all three methods used to quantify Pcy (microscopy and FCM providing absolute abundances, and 16S rRNA gene datasets providing relative abundances), and also consistent with 10 years of monitoring data, a pronounced seasonality of colonial Pcy/Syn ASVs was observed that followed peak abundances of single-cell Pcy/Syn ASVs and *Nostocales*. Previous studies from the Baltic Sea have reported contributions of colonial Pcy to the total Pcy population ranging from 5% to 70% [21, 38, 54, 55]. Two studies report high densities of total Pcy in early June and peak abundance of colonial/aggregating Pcy in early July [38, 55], and one report observed colonial Pcy increasing (from 5% to 70% of total Pcy biomass) in postbloom samples compared to samples collected during a *Nostocales* bloom [21]. In the southern Baltic Sea, high densities of total Pcy were observed prior to the *Nostocales* bloom in 2018 [53], followed by a shift in Pcy ASV composition during summer [33]. Our data, together with these other reports, suggest that the observed successional pattern of single-cell Pcy dominance in early summer prior to or concurrent with the *Nostocales* bloom, followed by an increase in colonial Pcy, is a consistent and important phenomenon for the Baltic Sea.

From microscopic observation, the colonial Pcy constitute a major component of the phytoplankton community. Based on this fact, we conclude that the three dominant colonial morphotypes must genetically be represented among the identified Syn ASVs, and thus belong to the *Synechococcus/Cyanobium* clade. This conclusion agrees with previous phylogenetic analyses of Baltic Sea Pcy [33] including of colonial morphotypes [49] and is also supported by the close match in our dataset between the microscopy determined seasonal occurrences of these morphotypes and the three most abundant colonial Syn ASVs. This congruency between independent measures provides support for the observed ASV size fraction enrichment reflecting aggregation/colony formation and also allows us to propose a genotype assignment of these organisms. Based on their ranked abundances, ASVs 2297, 2298, and 2329 would correspond to the Baltic Sea colonial Pcy morphotypes 1, 2, and 3 respectively. Further studies are, however, necessary to confirm this assignment and to what extent it warrants revision of the current morphology-based taxonomic classification of Baltic Sea colonial Pcy. For a more detailed discussion on the genetic versus morphology-based taxonomy, see Supplementary information.

Colony formation/aggregation in Pcy can be induced by environmental factors such as grazing and nutrient limitation [25, 56]. Recent studies have also shown that the “colonial” Pcy taxa *Cyanodictyon* and *Aphanothece* in their natural environment exist as both single cells and colonies [27, 49]. Although some work suggests that Pcy in the Baltic Sea are nitrogen (N) limited [53, 57] and depend on fixed N from *Nostocales* [34, 58], phosphate (P) levels typically decline during the *Nostocales* bloom [59, 60] and P limitation may occur 2–4 weeks postbloom [61]. High abundances of colonial Pcy have been reported from P-limited waters [62], and rates of N (ammonium and urea) uptake are reduced in colonial Pcy compared to single-cell Pcy [38]. Even though further investigations are required to understand the observed succession pattern, the differences in temporal abundance of colonial and single-cell ASVs, combined with the fact that colonial and single-cell ASVs appear not to form monophyletic groups, suggest that colony/aggregate formation is an important trait for determining distribution patterns and response of Pcy strains to environmental conditions.

### Sinking of Pcy and other phytoplankton

*Synechococcus* dominated among phytoplankton 16S rRNA gene sequence reads not only in surface waters but also in sediment traps and at 75 m depth, suggesting that relative to other phytoplankton groups, Pcy constitute an important component of sinking phytoplankton-derived particulate C during summer months in the brackish Baltic Sea. These findings agree with previous reports from the oligotrophic open ocean of *Synechococcus* contributing significantly to C export [7, 10, 12–14, 16, 17, 63] and suggest that such relevance of *Synechococcus* in C export may also apply to coastal and temperate waters.

In sedimentation of phytoplankton, cell size is an important determinant [18], and for small-enough cells such as Pcy, the sinking velocity is considered negligible. Given the fact that a major component of the Pcy population exist as single cells our finding that these organisms (i.e. colonial and single cells combined) sink less than the larger-sized eukaryotic phytoplankton and *Nostocales* is expected. The effect of size is further emphasized by sinking (i.e. trap representation) largely correlating with the large size fraction enrichment (i.e. Syn < Chloroplasts < *Nostocales*). Size fractionation is a common method to investigate differences between large and/or particle attached and small/free-living plankton [64–66]. The observed depth dependent increase in both large fraction Syn representation and percent colonial cells suggests that Pcy in these waters are, to a significant extent, sedimenting as colonies or aggregates. This conclusion agrees with earlier studies from the Sargasso Sea, North Atlantic, Southwest Pacific, and the Baltic Sea that reported Pcy cells in sinking detrital aggregates [9, 67–69].

### Sinking of Syn ASVs

Our finding that Syn ASVs over the sampling season show consistent differences in their representation in traps, 75 m samples and also in the large size fraction, suggest that both the propensity to sediment through the water column and the tendency to form colonies differ among Baltic Sea Pcy strains. We find a robust linear relationship between ASV large size fraction representation and ASV trap representation, suggesting that the extent to which Syn ASVs sediment through the water column is directly determined by their degree of colony formation and/or size of their colonies. Differential sedimentation among Pcy has been documented previously, for example, identifying *Synechococcus* as more prone to sink than *Prochlorococcus* [10, 12, 14, 16, 17]. There are also some recent reports on differential sinking among *Synechococcus* strains [13, 14]. Mechanisms proposed to explain these observations included differential grazing pressure, digestion resistance, and aggregation properties, but no direct observations were made to support these hypotheses.

Colony formation could contribute to sedimentation either by direct gravitational settling of colonies or via ingestion by mesozooplankton and subsequent sedimentation of fecal pellets [18, 19, 70, 71]. It is also possible that the extracellular matrix of colonies makes them more “sticky” and thereby prone to associate with other (sinking) phytoplankton, cell debris, or detrital particles. The effect of colony formation and aggregation on sedimentation rates are well documented for larger phytoplankton including chain and aggregate-forming diatoms [72–74]. For Pcy, aggregation and/or colony formation is a commonly assumed mechanism allowing biomass export [6], but few direct observations have been reported. Laboratory experiments demonstrated an increased settling rate for *Synechococcus* after induced aggregation [24], and in the Sargasso Sea, the relative abundance of Pcy pigments in large size fraction (>10 μm) was observed to increase with depth (down to 500 m) [9]. However, there are no previous reports correlating *in situ* quantifications of photic zone Pcy aggregates/colonies with sedimentation below the sunlit layer.

In our sampling scheme, discrete water samples were collected at the same depth as the sediment traps (25 m) and additionally at 75 m. Whereas sediment traps primarily collect sinking particles, water sampling may also collect suspended material exported to depth by convective mixing. However, the 75 m samples were collected well below the photic zone (10–16 m), as well as the mixed layer (5–25 m) and phytoplankton detected at 75 m therefore likely represent gravitational settling of cells or cell debris. The observed depth dependent temporal succession of abundance maxima of the dominant colonial Syn ASV2297 further suggests that the DNA sequences detected at depth represent recently sunk or sinking biomass originating from surface waters. Based on theoretical calculations (i.e. Stokes law) [75] and experimental data [18], the sinking rate estimated for ASV2297 (3.6 m/day), would be expected for a cell of ~20–50 μm in diameter, which is consistent with the size range of Pcy colonies in the Baltic Sea. *Aphanizomenon* not only forms large colonies (several millimeters) but also possesses gas vacuoles for buoyancy regulation and may largely decompose in surface layers [76]. The low sinking rate observed for *Aphanizomenon* is consistent with sedimentation derived from cell debris rather than intact cells [77], whereas a higher sinking rate for the large *Coscinodiscales* diatoms (50–370 μm) agrees with previously reported sedimentation velocity of senescent *Coscinodiscales* cells (10–50 m/day, [18]).

Phytoplankton biomass can either be exported directly via sinking of cells or aggregates or indirectly by grazing and subsequent sinking of fecal pellets. Given that fecal pellet sinking rates are usually much higher (30–1000 m/day; [78]) than those estimated here our results could suggest that the primary export route of colonial Pcy is via direct sinking of aggregates. However, other studies have suggested that picoplankton biomass is exported mainly via grazing on picoplankton aggregates and sinking as fecal pellets, and only to a lesser extent (10%) via direct sinking of aggregates [6]. Possibly, fecal pellets were under-sampled in our 75 m water samples, which indeed would be expected given the small sampling volumes collected [9].

## Conclusion

Picophytoplankton have been suggested to contribute significantly to C export [6–16], but the mechanism responsible for this export has not been determined. It has also been unclear why certain picophytoplankton contribute more than others to this process. Here we have shown that aggregation/colony formation among certain picocyanobacterial strains in the Baltic Sea allow for a greater proportion of their biomass being exported compared to strains that exist as single cells. We also find colony formation to be a variable trait among Baltic Sea Pcy strains, with clear seasonal succession patterns between colonial and single-cell Pcy strains and N_2_-fixing *Nostocales*, that are possibly driven by nutrient dynamics. Together these findings suggest an important role of Pcy colony formation/aggregation both in terms of ecology and biogeochemical C cycling and highlight the need for continued investigations on the prevalence and regulation of such morphotypes in other oceanic provinces.

## Supplementary Material

Supplementary_material_wrag214

## Data Availability

The sequence data underlying this article are available in NCBI (https://www.ncbi.nlm.nih.gov) and can be accessed with BioProject ID PRJNA1184790 and Biosample Accession Nos SAMN44675630-715. Other data underlying this article are available in the article and in its online supplementary material.
