# Supplementary material for: Differential aggregation properties among Baltic Sea picocyanobacterial strains impact their export
Source: ISME J. 2026 Aug 27;20(1):wrag214. doi: 10.1093/ismejo/wrag214 (PMC13630569; doi:10.1093/ismejo/wrag214)
Supplement: Supplementary file 1 [file supplementary_material_wrag214.zip › Supporting information.pdf]

-

Supporting Information for

Differential aggregation properties among Baltic Sea 3 picocyanobacterial strains impact their export

Martin Ekman[1, 2, *], Elina Viinimäki[1, 5], Anders Svensson[1], Ryan W. Paerl[4], Anders F. Andersson[3], Helena Höglander[1], Jakob Walve[1], Rachel A. Foster[1]

*Correspondence: Martin Ekman
Email: martin.ekman@regionstockholm.se

This PDF file includes:

Supporting Methods
Supporting Discussion
Figures S1 to S10
Tables S1 and S2

SUPPORTING METHODS

Flow cytometry analysis of picocyanobacterial abundance - using the FCM counts to calculate total and single cell Pcy cell concentrations and biomass

From microscopy analysis of water samples collected in August 2022 the average number of cells per colony was determined to 50. Assuming spherical colonies, and loosely packed cells this number is compatible with the average forward scatter determined colony diameter (6 µm) of samples collected for the present study (2018). Theoretically 123 spheres of 1 µm in diameter can be packed in a sphere with 6 µm in diameter (1). To recalculate colony counts to cell counts a conversion factor of 50 was therefore used. For microcolonies a recalculation factor of 2 was used. Total cell concentration was subsequently calculated by adding the colonial and microcolonial cell counts (based on fluorescence and forward scatter) to the total counts (based on fluorescence only). Colony counts obtained from FCM was considerably lower than those obtained from microscopy (Fig. S2c), which indicate that some colonies were broken up into single cells during FCM analysis. Single cell concentrations were therefore determined by subtracting the microscopy determined colonial cell concentrations from the flow cytometry determined total cell concentration. Microcolonies were thus categorized as single cells rather than colonies, which agree with the microscopy-based colony counts (see main manuscript method section). For calculating biomass from FCM counts, the average cell diameter determined for single cells (1.01 µm) was used (2).

Comments on combining abundances from size fractions to obtain an approximation of non-size fractionated abundances for the 02 July DI sample

Dupont et al (2015) (3) described a method to combine data from size fractions that had been analyzed with shotgun metagenomics. Here we applied this method to 16S rRNA gene sequence data from the two serial size fractions of the 2July DI sample, for which the non-size fractionated sample was lost. The data we obtained from this procedure is potentially biased since the 16S rRNA primers used, target bacteria and chloroplasts but not zooplankton. Given the size of most zooplankton this likely would lead to the

phytoplankton contribution to the large size-fraction being over-estimated. However, given that the large size fraction contributed with usually less than 10% of total DNA in the water samples we argue that the bias potentially introduced is likely small. Furthermore, when comparing the relative abundances of the dominant colonial ASVs in the two size fractions (Fig. S10a-c), the similarity suggests that combining these fractions results in a good approximation of abundances in a non-size fractionated sample.

References

1. J. Zhou, S. Ren, K. He, Y. Liu, C.-M. Li, An efficient solution space exploring and descent method for packing equal spheres in a sphere. Comput. Oper. Res. 164, 106522 (2024).
2. Menden-Deuer, S. and Lessard, E.J. Carbon to volume relationships for dinoflagellates, diatoms and other protist plankton. Limnol. Oceanogr. 45: 569-579 (2000).
3. C. L. Dupont, et al., Genomes and gene expression across light and productivity gradients in eastern subtropical Pacific microbial communities. ISME J. 9, 1076–1092 (2015).

SUPPORTING DISCUSSION

Taxonomy of colony-forming picocyanobacteria

In the Baltic Sea the phytoplankton community has been monitored for 30+ years using microscopy (morphological) based phytoplankton identifications. As described in the method section, such monitoring data was used in the present study to obtain abundances of colonial picocyanobacterial (Pcy) in surface waters. According to this data the dominant colonial morphotypes were morphologically classified to three genera *Cyanodictyon, Aphanocapsa*, and *Aphanothece*. As no16S rRNA gene sequences were annotated to these genera combined with the pronounced abundance of colonial Pcy in the water column, we conclude that the colonial Pcy (morphologically identified to *Cyanodictyon, Aphanocapsa*, and *Aphanothece*) must be represented among the

sequences annotated to *Synechococcus/Cyanobium* (i.e. Syn ASVs). This conclusion is supported by several previous studies as outlined below.

Huber et al (2017) [1] investigated Pcy colonies morphologically identified as *Cyanodictyon*. Colonies were individually sorted using flow cytometry and analyzed by 16S rRNA gene sequencing. All colonies were genetically classified as *Synechococcus* or *Cyanobium* (Huber et al 2017; Table S3). Albrecht et al (2017) [2] reported high abundances of Pcy colonies in the southern Baltic Sea. Based on morphology the colonies were identified as *Aphanothece*, but genetically (16S rRNA gene sequences) only *Cyanobium* was detected, and no sequences were annotated to *Aphanothece*. Komarek et al (2011) [3] used 16S rRNA gene sequencing of strains morphologically classified as *Aphanothece*, and found that this genus genetically consists of two distinct clusters; one belonging to Chroococcales and the other (renamed to *Anathece*) to the *Synechococcus/Cyanobium* phylogenetic clade. "*Anathece*" strains shared >96.8% sequence similarity with *Cyanobium gracile* which suggest they belong to the same genus (>95% sequence identity). The Komarek et al (2011) [3] results align with our phylogenetic analysis (Fig. 5), where the "Chroococcales-type" *Aphanothece* reference sequence cluster outside the *Synechococcus/Cyanobium* clade.

Together these previous studies confirm that the *Synechococcus/Cyanobium* clade contains members that form colonies, and these colonies are morphologically similar to the morphology based description of the genera *Aphanothece* and *Cyanodictyon*. These facts provide solid support for our conclusion that the colonial Pcy in our samples are represented among the Syn ASVs that we investigate.

Finally, given that no sequences in our dataset were annotated to *Cyanodictyon, Aphanocapsa,* or *Aphanothece*, we investigated the presence of sequences annotated to these genera in the Silva database. *Cyanodictyon* was originally described in 1914. No 16S rRNA gene sequence is available for the reference strain. In the Silva database, two sequences have the name *Cyanodictyon*. One of these originates from a salt flat in Oklahoma, USA, and is most closely related to *Dactylococcopsis* PCC-8305 within the order Cyanobacteriales. The second sequence originate from a freshwater reservoir in the

Czech Republic and is most closely related to *Cyanobium* PCC-6307 within the order Synechococcales. *Aphanothece* was originally described in 1849. The 16S rRNA sequence of the reference strain (*Aphanothece microscopica*) is available and classified within Chroococcales/Microcystaceae [4, 5]. Among the 33 "Aphanothece" sequences present in the Silva database, 20 belong to Cyanobacteriales, nine to Synechococcales, two to Leptolyngyales, one to Pseudanabaenales and one to Gloeobacteriales. There was no sequence annotated to *Anathece* in the Silva database. *Aphanocapsa* was originally described in 1849. No 16S rRNA gene sequence is available for the reference strain. Among the 20 "Aphanocapsa" sequences present in the Silva database 12 belong to the order Cyanobacteriales, three to Synechococcales, three to Thermosynechococcales and two to Phormidesmiales. The observed high phylogenetic diversity among the Silva database entries highlights that morphological classification of colonial Pcy may lead to miss-classification from a phylogenetic perspective, supporting our choice to rely on sequence-based classification.

References

1. Huber P, Diovisalvi N, Ferraro M *et al.* Phenotypic plasticity in freshwater picocyanobacteria. *Environ Microbiol* 2017;**19**(3):1120–33. https://doi.org/10.1111/1462-2920.13638.
2. Albrecht M, Pröschold T, Schumann R. Identification of Cyanobacteria in a Eutrophic Coastal Lagoon on the Southern Baltic Coast. *Front Microbiol* 2017;**8**:923. https://doi.org/10.3389/fmicb.2017.00923.
3. Komárek J, Kaštovský J, Jezberová, J. Phylogenetic and taxonomic delimitation of the cyanobacterial genus *Aphanothece* and description of *Anathece* gen. nov. European Journal of Phycology, 2011;46(3), 315–326.
4. Komárek J, Kaštovský J, Mareš J, Johansen JR. Taxonomic classification of cyanoprokaryotes (cyanobacterial genera) 2014, using a polyphasic approach. Preslia 2014; 86:295-335.
5. O. Strunecký, A. P. Ivanova, J. Mareš, An updated classification of cyanobacterial orders and families based on phylogenomic and polyphasic analysis. J. Phycol. 2023;59, 12–51.

SUPPORTING FIGURES

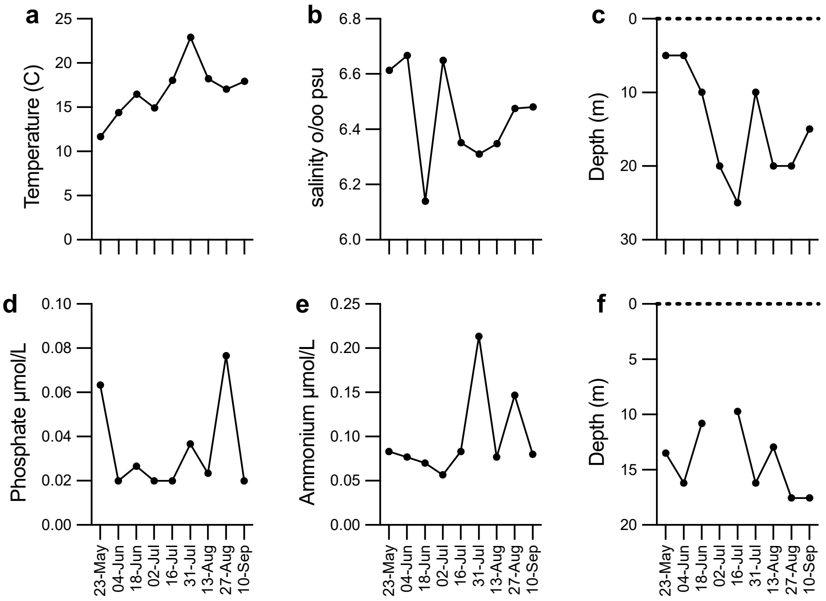

Fig. S1. Hydrographic data. Temperature, salinity, phosphate and ammonium are average values of water samples collected from 0m, 5m and 10m. **a.** Temperature: **b**. salinity **c**. Mixed layer depth. **d**. Phosphate **e**. Ammonium **f**. Photic zone depth (missing value 02-July). Analytical quantification limits for phosphate and ammonium were 0.016 and 0.036 µmol/L, respectively.

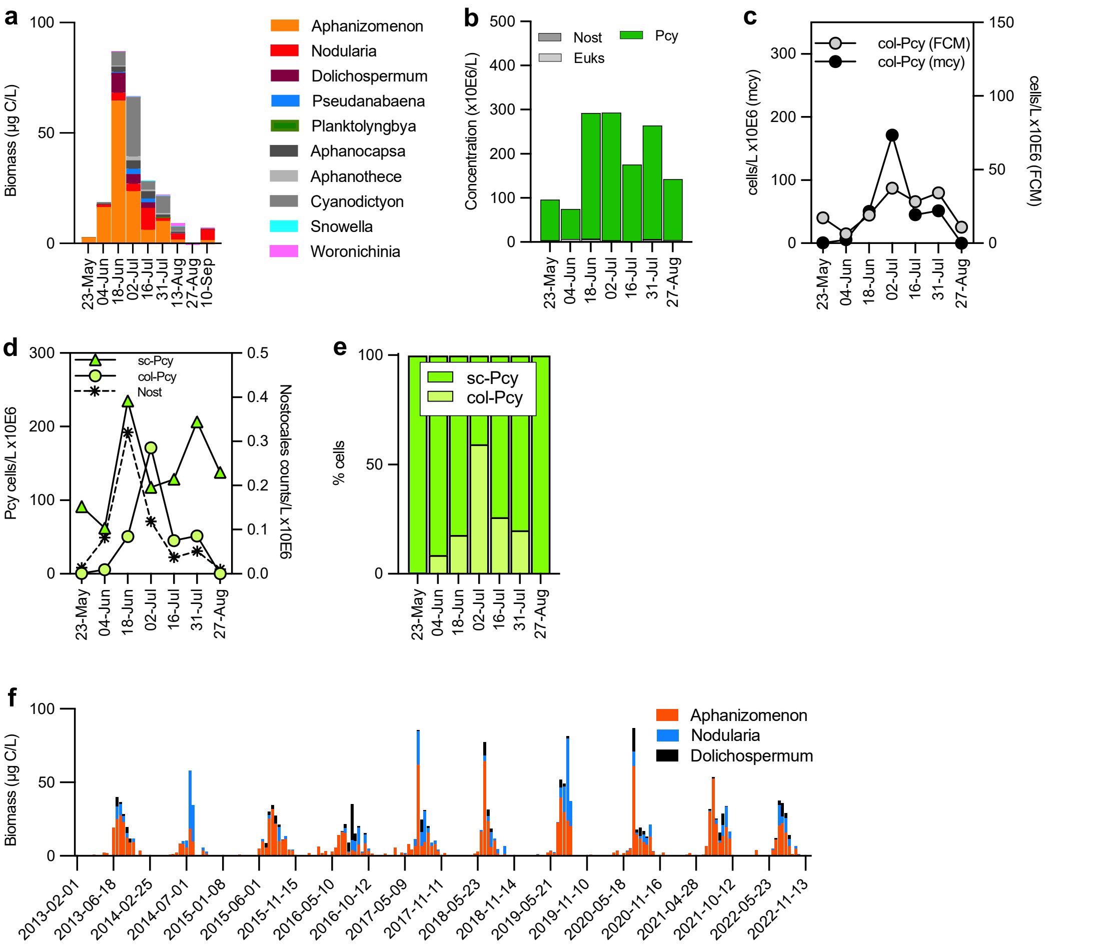

Fig. S2. Seasonal trends in Baltic Sea phytoplankton at finer taxonomic resolution (a and f) and as cell concentrations instead of biomass (b-e). a. Biomass of all cyanobacteria identified via microscopy. Note that these does not include single cell Pcy. Colonial Pcy genera are *Aphanocapsa* (Morphotype 2), *Aphanothece* (Morphotype 3), and *Cyanodictyon* (Morphotype 1) and Nostocales genera are *Aphanizomenon, Nodularia and Dolichospermum.* **b**. Concentration of eukaryotic phytoplankton (Euks), Nostocales (Nost) and picocyanobacteria (Pcy). Plotted values are cell concentrations for Pcy and Euks and concentration of 100 µm pieces for Nostocales. Picocyanobacteria represent combined values for colonial Pcy (col-Pcy) and single cell Pcy (sc-Pcy). **c.** Comparison of concentration of colonial Pcy determined by FCM and microscopy. **d-e**. The same data as in Figure 1b-c but reported as cell concentration (for Pcy) or concentration of 100 µm pieces (for Nostocales). **f**. Biomass of Nostocales genera 2013-2022.

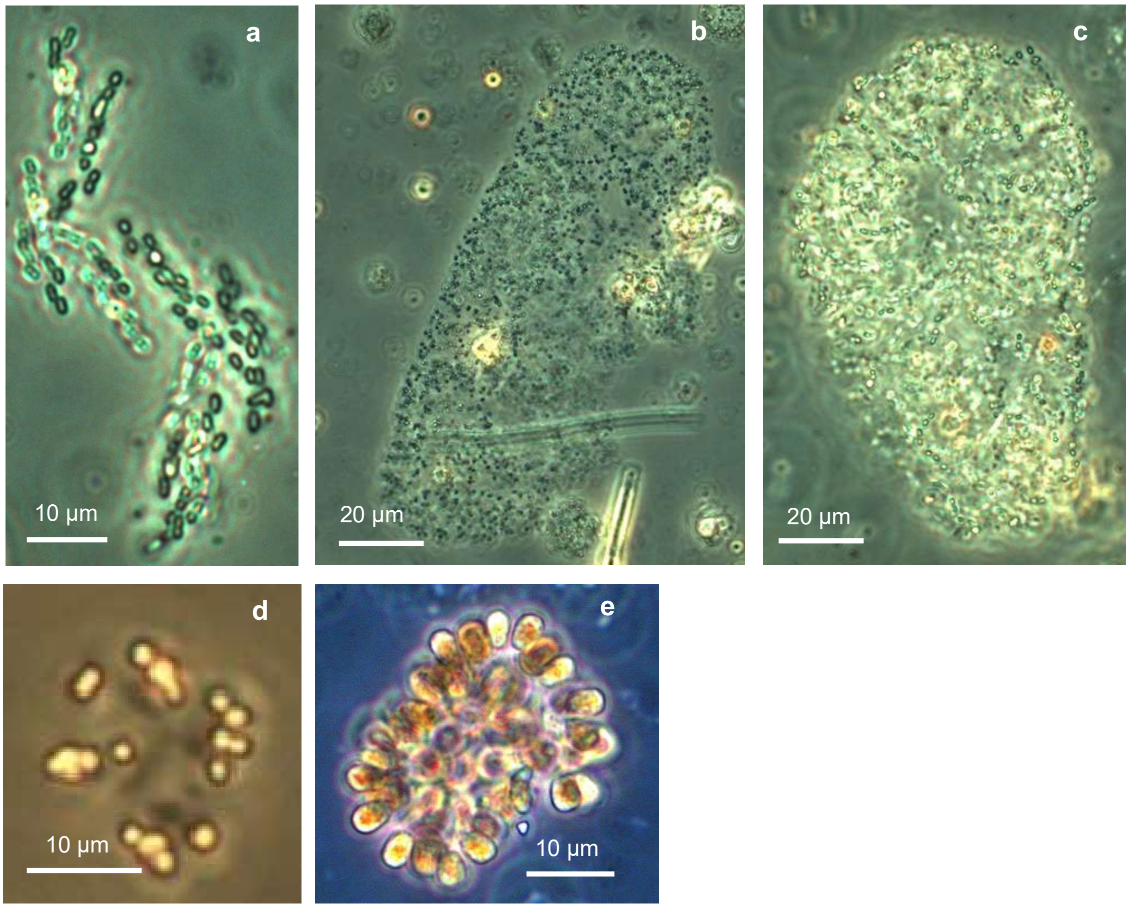

Fig. S3. Colony forming picocyanobacteria at BY31 (a-c) and colony forming cyanobacteria with larger cell size (1-5 µm) (d-e): **a**. *Aphanothece paralleliformis* (Morphotype 2) with cells in flat colonies and cylindrical-shaped cells (1.5-2.0 x 0.8-1.0 µm) in rows. **b**. *Aphanocapsa* sp. (Morphotype 3) with round cells (1.2 µm in diameter) in spherical or irregular colonies with cells also inside the colony and **c**. *Cyanodictyon* sp (Morphotype 1) with oval cells (1.0 x1.2 µm) in spherical or irregular colonies with cells also inside colony. In some species cells are arranged in netlike structures. **d**. *Snowella* sp. round cells (1-4 µm) on thin gelatinous stalks, in loose to more compact colonies **e**. *Woronichinia* sp. oval cells (2-5 µm) on gelatinous stalks in compact round to irregular hollow colonies.

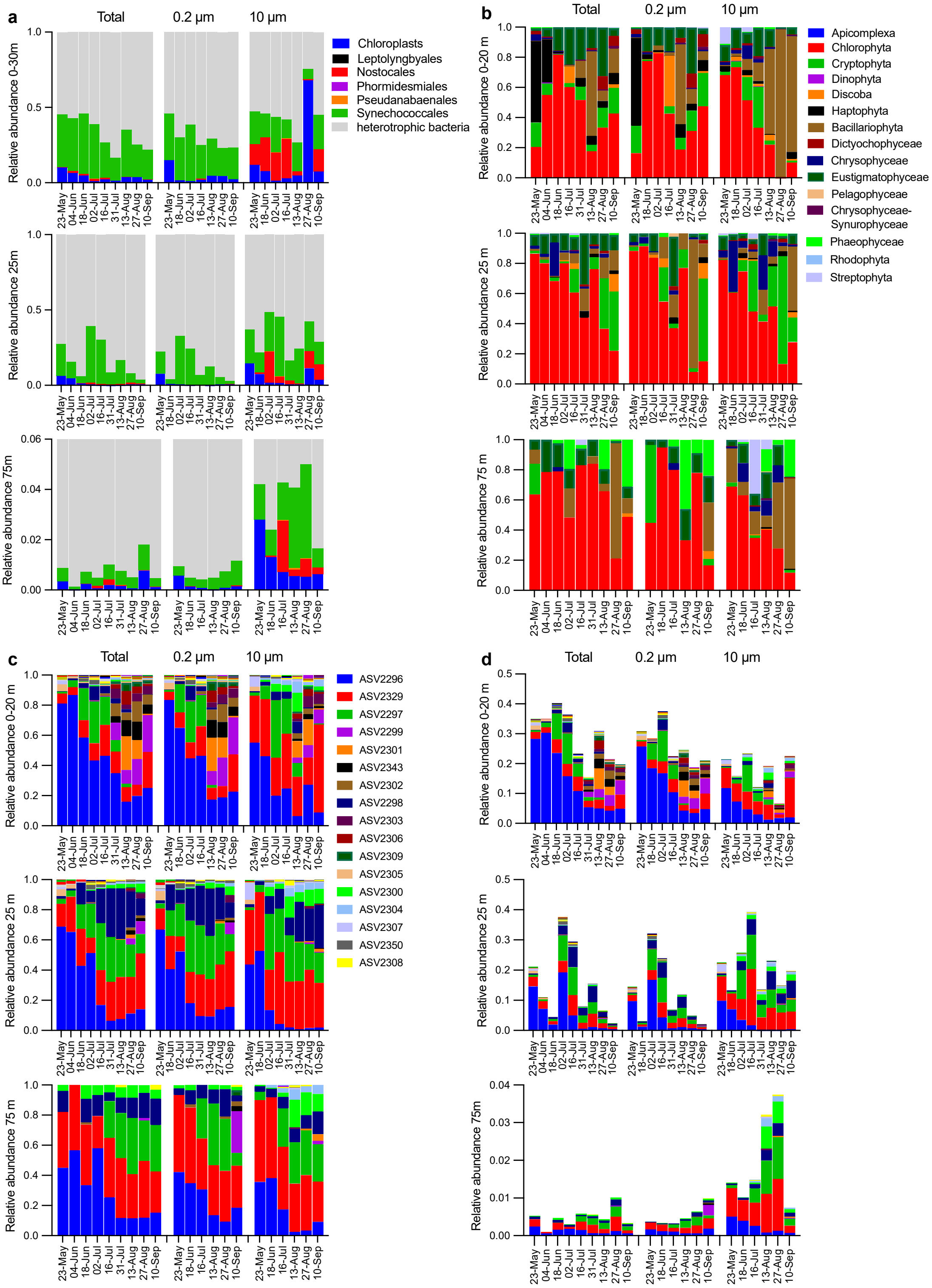

Fig. S4. 16S rRNA based relative abundance of all collected water samples and traps (note different scales in diagrams). **a.** Over-all phytoplankton community. 'Chloroplasts' indicate eukaryotic phytoplankton. **b.** Eukaryotic phytoplankton. **c.** Syn ASVs as fraction of total Syn reads. **d**. Syn ASVs relative all reads.

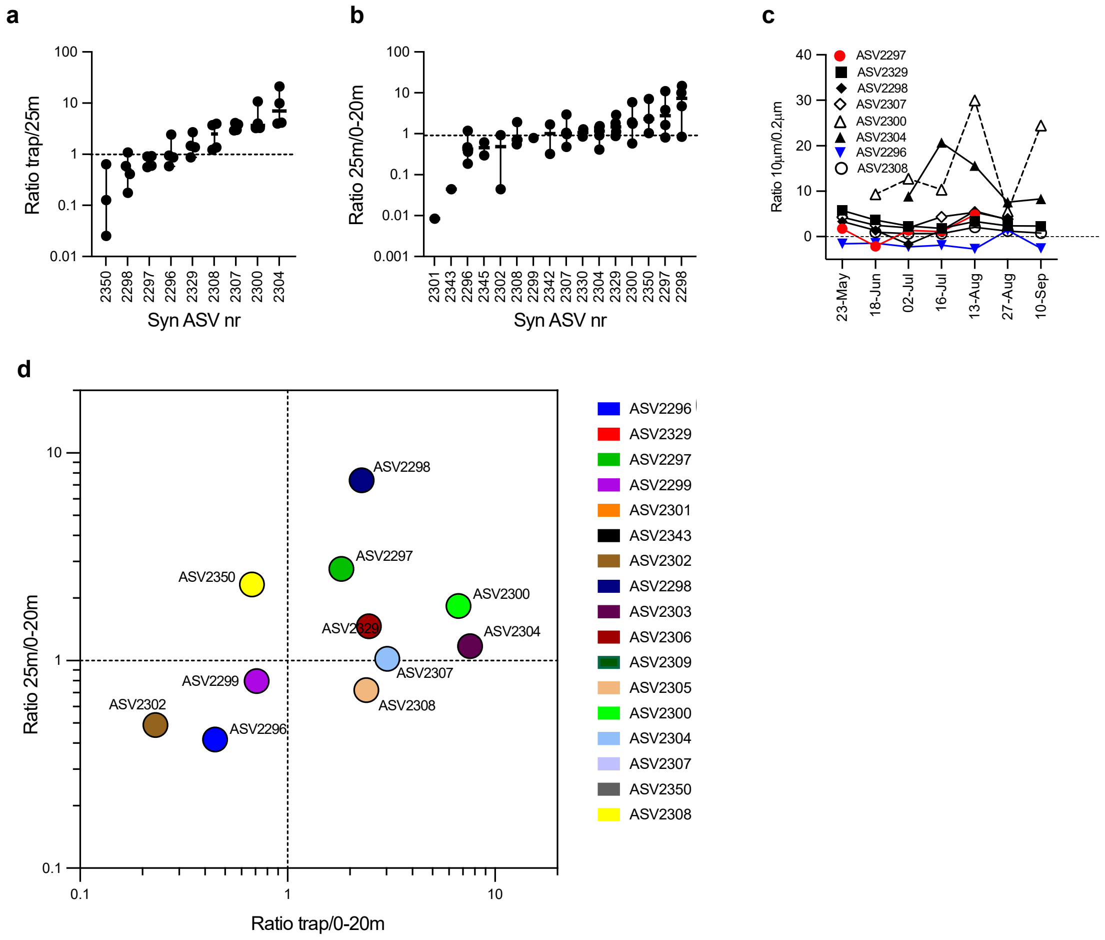

Fig. S5. Representation of Syn ASVs in 25 m samples (a-b, and d) and in the large size fraction of 0-20m samples (c). **a.** Ratio between rel. abundance in traps and in 25 m waters. **b.** Ratio between relative abundance in 25 m samples and DI samples. **c.** 10 µm/0.2 µm ratio for colonial Syn ASVs and the single cell ASV2296 in 0-20 m non-size fractionated samples over the season. **d.** Correlation between ratio 25m/0-20 m and trap/0-20m. R^2=0.023, p=0.619

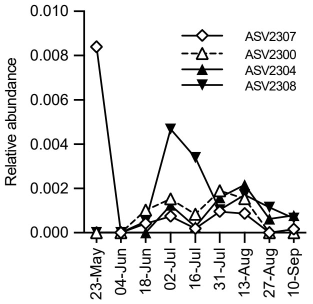

Fig. S6. Seasonal trends for colonial ASV:s of lower abundance in 0-20 m non-size fractionated samples.

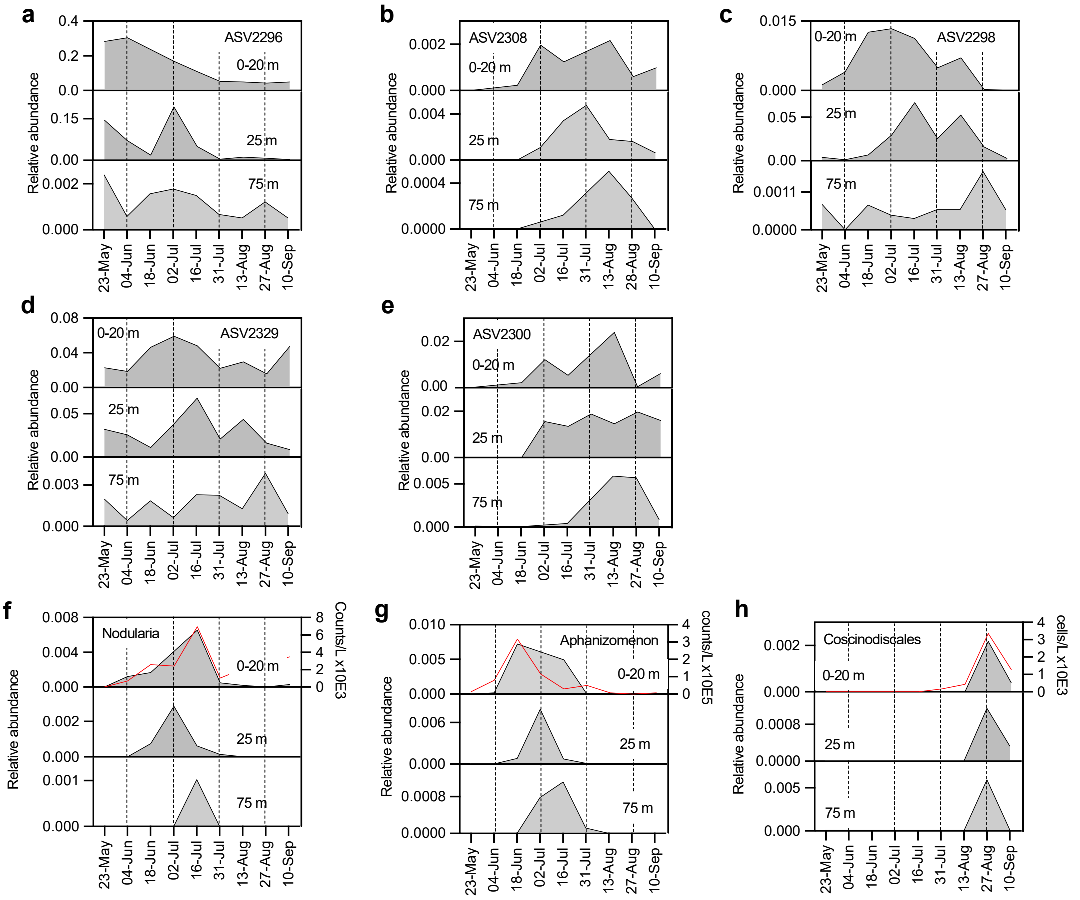

Fig. S7. Relative abundance at the three sampling depths of 16S rRNA sequences representing Syn ASVs (a-e), *Nodularia* (f), *Aphanizomenon* (g) and Coscinodiscales (h). The red line in (f-g) indicate the microscopy determined seasonal abundance (right axis; counts of 100 µm-pieces) of *Nodularia* and *Aphanizomenon.* The red line in (h) indicate the microscopy determined seasonal abundance (right axis; cell counts) of Coscinodiscales. All data from non-size fractionated samples except ASV2300 and ASV2308 that are from the 10µm size fraction.

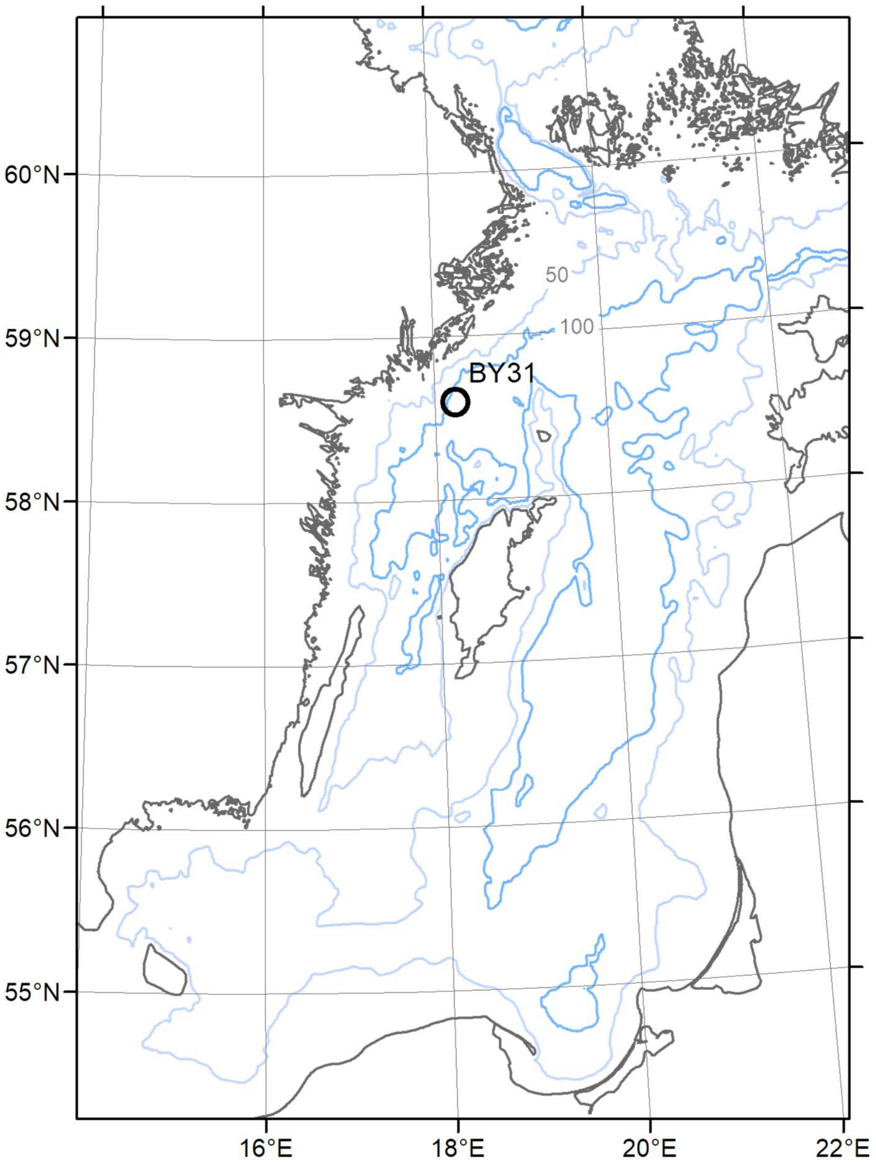

Fig. S8. Map of southern Baltic Sea with sampling station BY31 and depth curves in meters indicated.

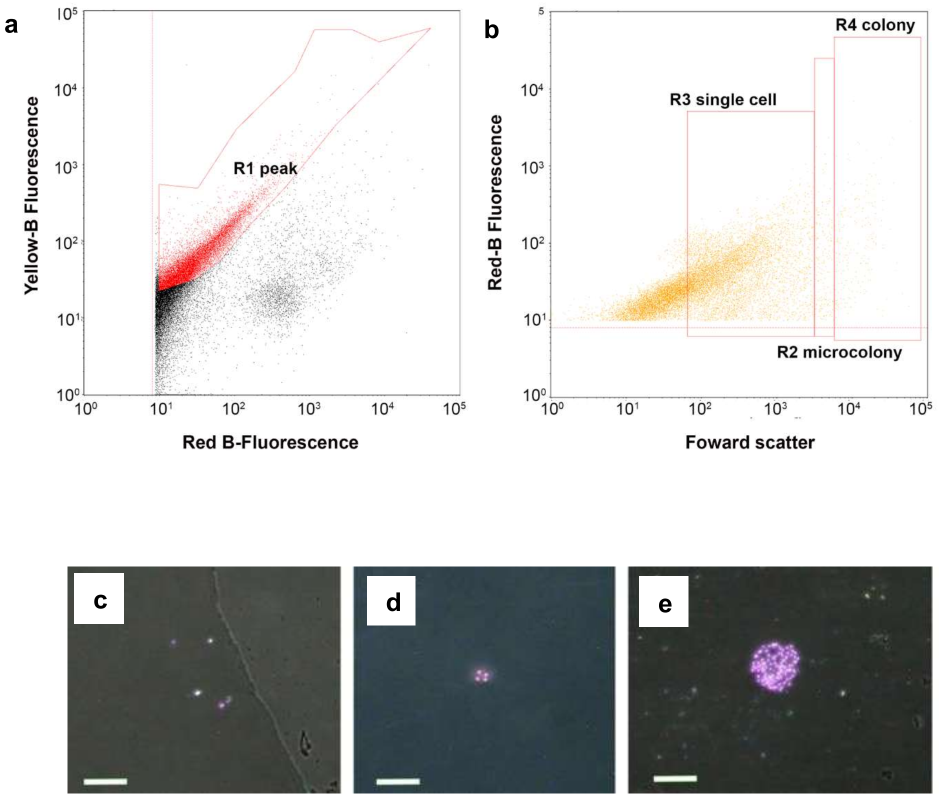

Fig. S9. Flow cytometry gating strategy. a-b. Gates used for separating single cells, microcolonies and colonies. **c-e.** examples of micrographs from the three sorted forward scatter FCM categories R3/single cells (c), R2/microcolonies (d) and R4/colonies (e). The scale bar is 10 µm.

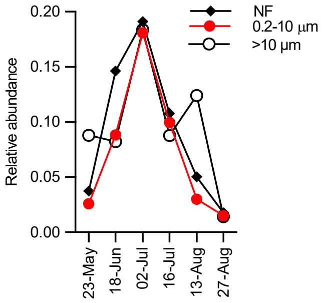

Fig. S10. Comparison of relative abundance of the colonial ASVs in the two size fractions (0.2-10 µm and >10µm) and in the non-size fractionated samples (NF).

Table S1. Summary of samples collected for DNA analysis (16S rRNA sequencing). Number of analysed samples are indicated together with number of collected samples in brackets. Sediment trap samples from brine and chloroform traps were not differentiated in the analyses. Dates are when samples were collected.

	Sediment trap samples		Water samples								
			0-20m			25m			75m		
Sample collection date	Chloroform	Brine	No size fractionation	0.2-10 *μ*m	>10 *μ*m	No size fractionation	0.2-10 *μ*m	>10 um	No size fractionation	0.2-10 *μ*m	>10 *μ*m
2018-05-23			1 (3)	1 (3)	1 (3)	1 (3)	1 (3)	1 (3)	1 (3)	1 (3)	1 (3)
2018-06-04			1 (3)			1 (3)			1 (3)		
2018-06-18			1 (3)	1 (3)	1 (3)	1 (3)	1 (3)	1 (3)	1 (3)	1 (3)	1 (3)
2018-07-02	1 (1)	1 (2)		1 (3)	1 (3)	1 (3)	1 (3)	1 (3)	1 (3)		
2018-07-16	2 (2)	2 (2)	1 (3)	1 (3)	1 (3)	1 (3)	1 (3)	1 (3)	1 (3)	1 (3)	1 (3)
2018-07-31	3 (3)	3 (3)	1 (3)			1 (3)	1 (3)	1 (3)	1 (3)		
2018-08-13	2 (2)	4 (9)	1 (3)	1 (3)	1 (3)	1 (3)	1 (3)	1 (3)	1 (3)	1 (3)	1 (3)
2018-08-27			1 (3)	1 (3)	1 (3)	1 (3)	1 (3)	1 (3)	1 (3)	1 (3)	1 (3)
2018-09-10			1 (3)	1 (3)	1 (3)	1 (3)	1 (3)	1 (3)	1 (3)	1 (3)	1 (3)

Table S2. Abundance of Syn ASVs in non-size fractionated DI samples shown as fraction of all Syn ASV reads. ND; not detected.

		23-May	04-Jun	18-Jun	02-Jul	16-Jul	31-Jul	13-Aug	27-Aug	10-Sept
"Single cell" ASVs	ASV2296	81,2	86,7	58,6	43,4	46,5	34,9	16,1	19,9	25,1
	ASV2299	ND	ND	0,1	0,4	0,3	11,4	9,2	17,1	24,5
	ASV2301	ND	ND	ND	ND	ND	ND	22,3	12,6	0,8
	ASV2343	ND	ND	ND	ND	ND	4,4	10,5	12,2	6,2
	ASV2303	ND	ND	ND	ND	ND	6,6	2,9	5,4	5,4
	ASV2302	ND	ND	0,1	0,4	1,8	8,5	5,2	13,4	6,7
	ASV2305	4,4	1,5	0,8	ND	0,8	ND	0,8	0,8	0,6
	ASV2306	ND	ND	ND	ND	ND	2,9	9,1	4,4	1,5
	ASV2350	ND	0,2	1,9	3,0	0,3	ND	0,4	0,0	0,0
	ASV2309	ND	ND	ND	ND	ND	1,8	5,3	3,2	2,4
"Colonial" ASVs	ASV2329	6,6	5,3	11,5	11,4	20,5	14,3	9,6	7,4	23,9
	ASV2297	1,3	4,5	21,2	27,8	18,7	7,7	2,2	ND	ND
	ASV2298	0,4	1,2	3,1	9,2	4,8	3,2	2,3	0,1	0,0
	ASV2300	ND	ND	0,3	0,7	0,4	1,2	0,5	ND	ND
	ASV2304	ND	ND	ND	0,5	0,1	1,0	0,7	0,3	0,4
	ASV2307	2,4	ND	0,1	0,2	0,1	0,6	0,3	ND	0,1
	ASV2308	ND	ND	0,1	1,2	1,5	0,7	0,6	0,5	0,3
	Sum colonial ASVs	10,6	11,0	36,4	50,9	46,1	28,7	16,2	8,3	24,8
	Sum Single cell ASVs	85,6	88,4	61,6	47,1	49,6	70,6	81,8	89,0	73,3
